# Contents in tumor-educated platelets as the novel biosource for cancer diagnostics

**DOI:** 10.3389/fonc.2023.1165600

**Published:** 2023-04-17

**Authors:** Qianru Zhang, Xianrang Song, Xingguo Song

**Affiliations:** ^1^Department of Clinical Laboratory, Shandong Cancer Hospital and Institute, Shandong First Medical University and Shandong Academy of Medical Sciences, Jinan, Shandong, China; ^2^Shandong Provincial Key Laboratory of Radiation Oncology, Shandong Cancer Hospital and Institute, Shandong First Medical University and Shandong Academy of Medical Sciences, Jinan, Shandong, China

**Keywords:** tumor-educated platelets (TEPs), cancer diagnostics, mRNA, non-coding RNA, proteome

## Abstract

Liquid biopsy, a powerful non-invasive test, has been widely used in cancer diagnosis and treatment. Platelets, the second most abundant cells in peripheral blood, are becoming one of the richest sources of liquid biopsy with the capacity to systematically and locally respond to the presence of cancer and absorb and store circulating proteins and different types of nucleic acids, thus called “tumor-educated platelets (TEPs)”. The contents of TEPs are significantly and specifically altered, empowering them with the potential as cancer biomarkers. The current review focuses on the alternation of TEP content, including coding and non-coding RNA and proteins, and their role in cancer diagnostics.

## 1 Introduction

There has been remarkable progress in the field of cancer diagnostics; however, tissue biopsy remains the most important and only method of making a definitive diagnosis. Because tissue biopsy is traumatic and infeasible for serial collection, liquid biopsy has become a hot research direction with remarkable advances including non-invasive, easy-to-obtain, and real-time monitoring (1). At present, liquid biopsy mainly focuses on cell-free DNA (cfDNA) (2), circulating tumor cells (CTCs) (3), extracellular vesicles (EVs) (4), circulating tumor RNA (5), and, more recently, tumor-educated platelets (TEPs) (6, 7). All of these biological sources present are considered to be powerful reservoirs of cancer biomarkers, contributing to early diagnosis and treatment, as well as to precision cancer medicine.

**Figure 1:**
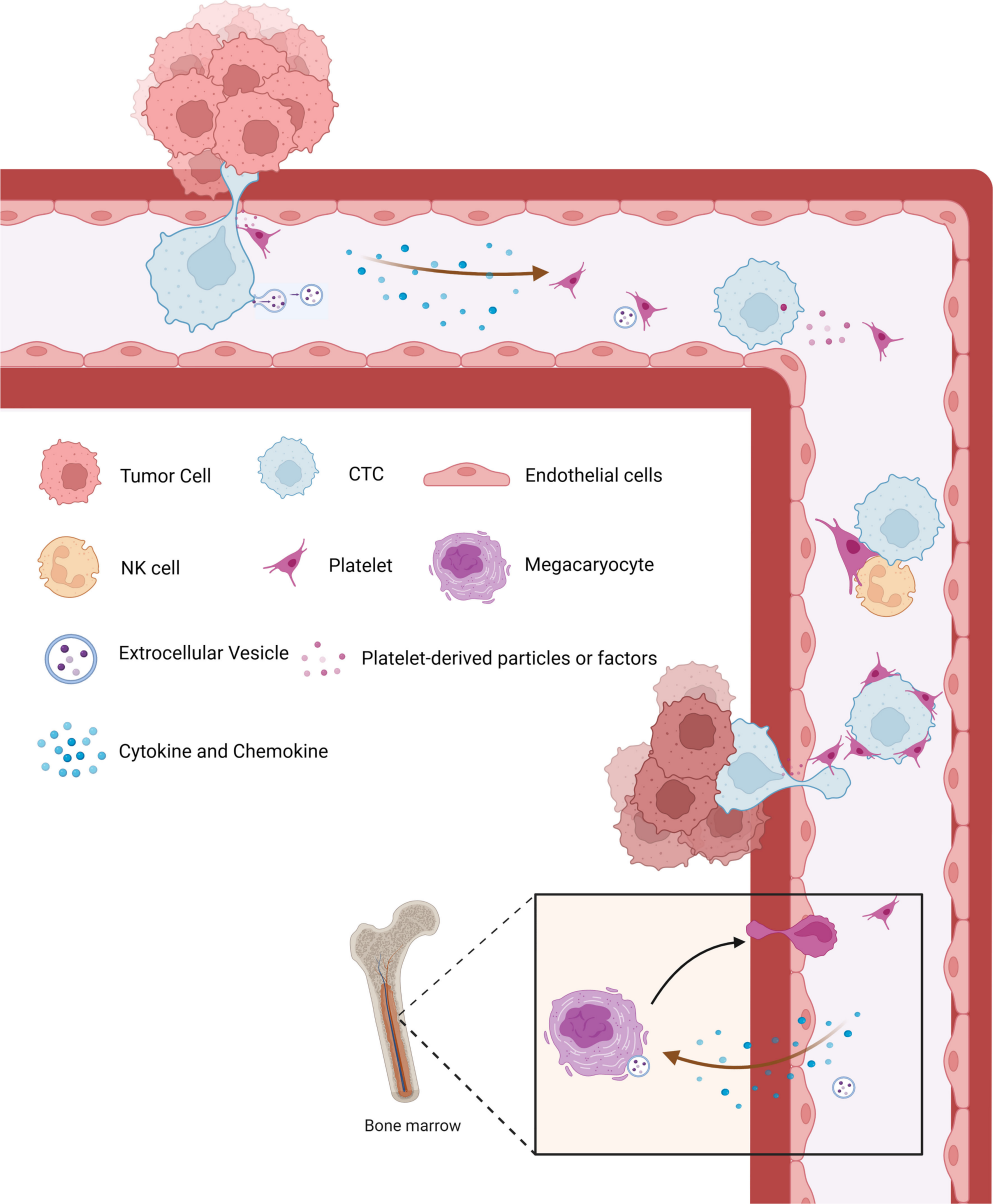
The crosstalk between cancer and platelets. Platelets are released into circulation by megakaryocytes and produce tumor-educated platelets through direct or indirect communication with tumor cells or tumor-derived biomolecules. Tumor cells can induce the activation and aggregation of platelets, which in turn secrete various factors that promote the growth and metastasis of the primary tumor. During metastasis, aggregated platelets protect CTCs from shear forces and evade immune surveillance. At the same time, platelets can recruit stromal cells to facilitate the establishment of metastatic niches and promote the metastasis of tumor cells. CTCs, circulating tumor cells.

Platelets, circulating fragments of anucleate cells originating from mature megakaryocytes (MKs), are the second most abundant cell type in peripheral blood with relatively short lifespans ranging from 8 to 11 days (8) and play a crucial role in hemostasis, thrombosis, and inflammatory processes (9–11). Over the past decades, multiple pieces of evidence indicate that platelets serve much more comprehensive functions in all steps of tumorigenesis, including tumor growth, tumor cell extravasation, angiogenesis, and metastasis (12). The interaction between platelets and tumor is the prerequisite for hematogenous metastasis (**Figure 1**). Platelets release many anti-angiogenic or pro-angiogenic factors when activated, which display the regulatory effect on vascular remodeling and vessel integrity, thus helping tumor cells adhere to and penetrate the endothelium (13). Upon arrival in the blood, tumor cells are covered and shielded by platelets from shear forces by lodging in the vessel wall (14), and they evade NK cells attack by impeding the immunologic recognition (15–17). Subsequently, platelets along with platelet-derived particles influence circulating tumor cells, leading to the transmission of mesenchymal-like phenotype, as well as capillary endothelium, to expedite extravasation in distant organs (18–20).

From another point of view, bidirectional tumor–platelet interactions are reciprocal and complicated on those platelets that enhance malignancies while tumors educate platelets (21–23). The education of platelets by tumor cells can be achieved in direct and indirect manners. In the bloodstream, straightforward contact occurs between molecules on platelets and tumor cells, including P-selectin (24–26), integrins (27, 28), and glycoproteins (29, 30), leading to platelet activation, so-called direct manner. Moreover, tumor cells can release metabolites extracellularly, including cytokines, chemokines, and, importantly, the extracellular vesicles, all of which serve as the indirect way to educate not only circulation platelets (31, 32) but also megakaryocytes in the bone marrow to subsequently alter platelet generation (33, 34) (**Figure 1**). Overall, platelets systematically and locally respond to cancer, absorbing and storing circulating proteins and different types of nucleic acids from the peripheral blood and tumor microenvironment (32), consequently sequestering tumor-specified biomolecules including RNA transcripts and proteins, which are called TEPs (7).

**Figure 2:**
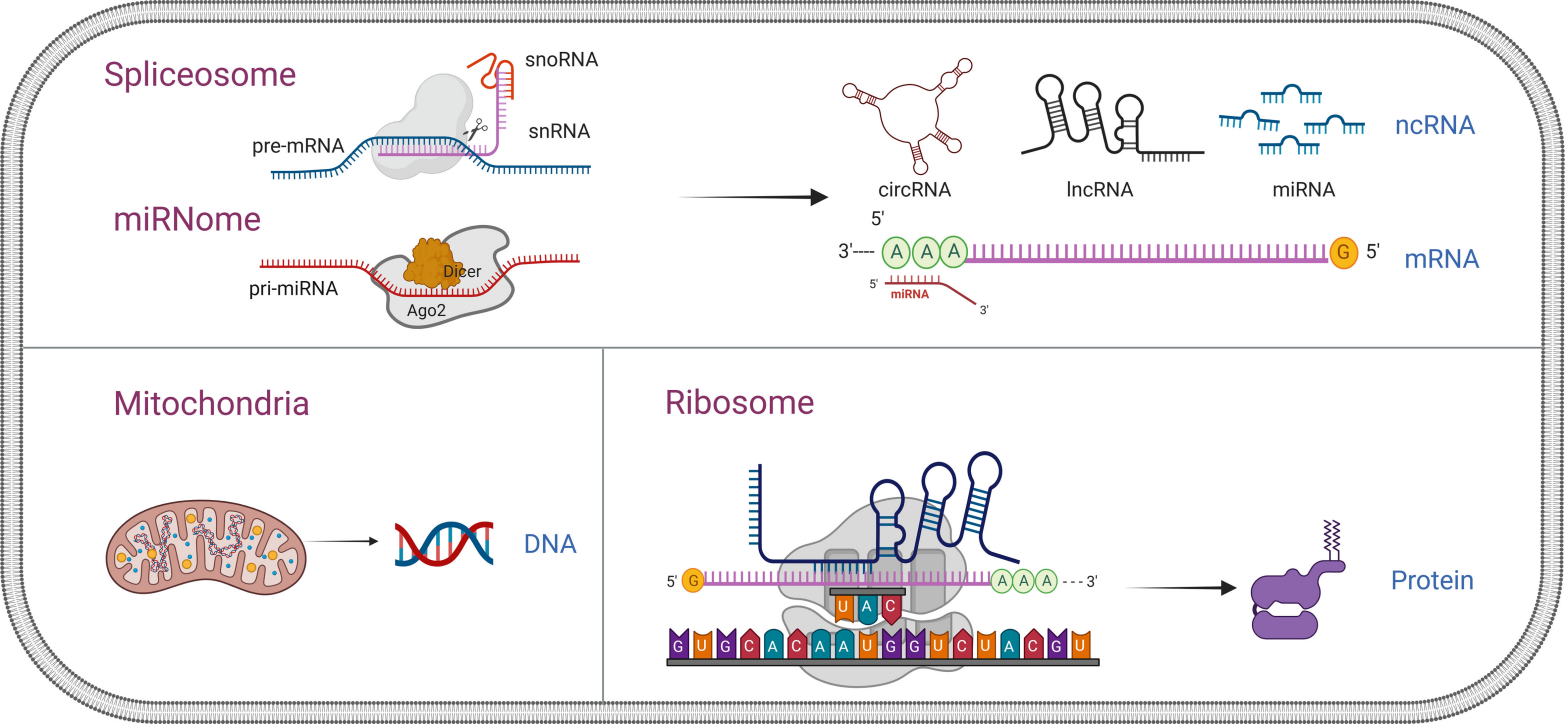
Comprehensive overview of nucleic acid and protein in platelets. Platelets lack the nucleus and thus possess no genomic but only mitochondrial DNA. They contain RNA molecules, including coding and non-coding, and proteins, which can be not only inherited from megakaryocytes but also generated in the platelets since platelets exploit functional spliceosome, ribosome, and other non-coding RNA (snRNA, snoRNA, miRNA, circRNA, and lncRNA) processing mechanisms. snRNA, small nuclear RNA; snoRNA, small nucleolar RNA; miRNA, microRNA; circRNA, circular RNA; lncRNA, non-coding RNA.

As high-throughput sequencing technology (35, 36) and computer identification algorithms (37, 38) have been developed in the past few years, the contents of platelets have been identified and well demonstrated. Platelets lack the nucleus and thus possess no genomic but mitochondrial DNA (39). They contain RNA molecules including coding and non-coding (40), and proteins (41), which can be not only inherited from megakaryocytes but also generated in platelets since platelets exploit functional spliceosome, ribosome, and other non-coding RNA processing mechanisms (42–44) (**Figure 2**). During tumor education of platelets, the contents in platelets are altered significantly and specifically in response to the presence of cancer, empowering them to serve as an important repository of potential RNA and protein biomarkers for early cancer detection (45), disease progression monitoring (7, 38), and response to treatment (46, 47).

**Figure 3:**
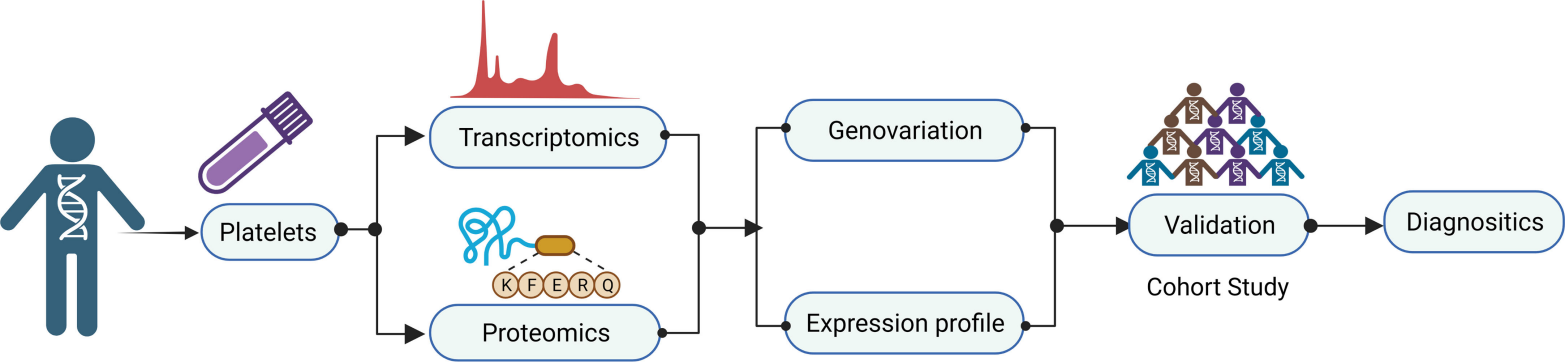
Workflow of tumor-educated platelet research for clinical applications. A typical workflow for studying TEPs as biomarkers in cancer consist of multiple steps. Platelet separation is the key step for the whole workflow because the platelets are fragile and easily activated in the environment. After separation, platelets are lysed for nucleic acids and protein extraction, which then are subjected to high-throughput sequencing or mass spectrometry to screen out the potential biomarkers and verified in a large-scale cohort. TEPs, tumor-educated platelets.

A typical workflow for studying TEPs as biomarkers in cancer, as shown in **Figure 3**, consists of multiple steps. Platelet separation is the key step in the whole workflow because platelets are fragile and easily activated in the environment. Currently, the most commonly used method of platelet separation is low-speed centrifugation. Anticoagulated whole blood is centrifuged at low speed to obtain platelet-rich plasma (PRP), followed by another centrifugation to precipitate platelets at room temperature (48). D’ambrosi et al. (49) used two methods to isolate platelets, one was conventional centrifugation and the other was adding Iloprost (50 nM) to PRP, both of which obtained the lowest activation and highest purity of platelets without significant differences. The standard for high-purity platelet preparation is less than 5 nucleated cells per 10 million platelets (37) and, more importantly, to avoid platelet activation. Detection of platelet activation markers contributes significantly to the quantitative control of platelet separation. After separation, platelets are lysed for nucleic acids and protein extraction, which are then subjected to high-throughput sequencing or mass spectrometry to screen out the potential biomarkers and verified in a large-scale cohort. In the current review, attention is paid to the alternation of contents in TEPs, including coding and non-coding RNA and proteins, and their role in cancer diagnostics (**Table 1**).

**Table 1:** Role of RNA molecules and proteins in cancer diagnosis in TEPs.

Marker type	TEP biomarkers	Tumor type	Expression	Test	Techniques	References
mRNA		Pan-cancer: NSCLC, CRC, GBM, PAAD, HBC, BRCA		Accuracy = 96%	RNA-seq, multiclass support vector machine (SVM)-based classification	(48)
	ITGA2B	NSCLC	Up	Test cohort: AUC = 0.922, validation cohort: AUC = 0.888	RNA-seq, q-PCR, ddPCR	(50)
	MAX, MTURN, HLA-B	Lung cancer	Up	AUC = 0.734, AUC = 0.787 (early lung cancer), AUC = 0.825 (MTURN mRNA as diagnostics biomarker for female lung cancer)	Microarray, q-PCR	(51)
	TIMP1	CRC	Up	AUC = 0.9583, TIPM1 mRNA carried into cancer cells by TEPs promotes cancer cell growth	RNA-seq, q-PCR	(52)
	TPM3	Breast cancer	Up	AUC = 0.9705 (diagnosis), AUC = 0.8404 (metastasis), platelet microvesicles from cancer patients promote cancer cell migration by delivering TPM3 mRNA	RNA-seq, q-PCR	(53)
	ACIN1	Lung cancer	Up	AUC = 0.608	q-PCR	(54)
MiRNA	MiR-34c-3p3p, miR-18a-5p	NPC	Up	AUC = 0.952 (miR-34c-3p3p), AUC = 0.884 (miR-18a-5p), AUC = 0.954 (combination)	q-PCR	(55)
	MiR-223	NSCLC	Up	Platelet miR-223 targeted EPB41L3 to promote A549 cell invasion		(56)
CircRNA	CircNRIP1	NSCLC	Down	p = 0.0302 (NSCLC), p = 0.0263 (late stage NSCLC), p = 0.098 (early-stage NSCLC)	RNA-seq, q-PCR	(49)
LncRNA	linc-GTF2H2-1, RP3-466P17.2, LCC-ST8SIA4-12	NSCLC		AUC = 0.781 (linc-GTF2H2-1), AUC = 0.788 (RP3-466P17.2), AUC = 0.725 (LCC-ST8SIA4-12), AUC = 0.921 (three lncRNA), early stage AUC = 0.704 (linc-GTF2H2-1), AUC = 0.771 (RP3-466P17.2), AUC = 0.768 (LCC-ST8SIA4-12), AUC = 0.895 (three lncRNA)	Microarray, q-PCR	(57)
	MAGI2-AS3, ZFAS1	NSCLC	Down	MAGI2-AS3 (AUC = 0.853, AD; AUC = 0.892, SCC); ZFAS1 (AUC = 0.780, AD; AUC = 0.744, SCC)	q-PCR	(58)
	LncRNA ROR	NPC	Down	Accuracy = 63.9%, AUC = 0.70	q-PCR	(59)
	LNCAROD, SNHG20, LINC00534, TSPOAP-AS1	CRC	Up			(60)
SnRNA	U1, U2, U5	Lung cancer	Down	AUC = 0.769 (U1), AUC = 0.840 (U2), AUC = 0.809 (U5), AUC = 0.840 (three snRNA); early stage AUC = 0.669 (U1), AUC = 0.805 (U2), AUC = 0.752 (U5), AUC = 0.826 (three snRNA)	q-PCR	(61)
SnoRNA	SNORD55	NSCLC	Down	AUC = 0.803 (NSCLC), AUC = 0.784 (early-stage NSCLC), AUC = 0.791 (LUAD), AUC = 0.759 (early-stage LUAD), AUC = 0.826 (LUSC), AUC = 0.854 (early-stage LUSC)	q-PCR	(62)
Protein	VEGF, PDGF, PF4	CRC	Up	AUC = 0.893	ELISA	(63)
	Platelet protein	OC		Late stage (III–IV): sensitivity = 96%, specificity = 88% Early stage (I–II): sensitivity = 83%, specificity = 76%, AUC = 0.831	Partial least squares discriminant analysis (PLS-DA)	(64
	Platelet count, MPV, and concentrations of VEGF, PDGF, PF4, CTAPIII, and TSP-1 in platelets and PFP	Lung cancer		AUC = 0.868	Multivariate modeling	(65)
	Platelet count, MPV, and VEGF concentration in platelets	Head of pancreas cancer		AUC = 0.827	Multivariate modeling	(65)

AUC, area under the receiver operating characteristic curve; NSCLC, non-small cell lung carcinoma; LUAD, lung adenocarcinoma; LUSC, lung squamous cell carcinoma; CRC, colorectal cancer; GBM, glioblastoma; PAAD, pancreatic cancer; HBC, hepatobiliary cancer; BRCA, breast cancer; ddPCR, droplet digital PCR; NPC, nasopharyngeal carcinoma; AD, adenocarcinoma; SCC, squamous cell carcinoma; OC, ovarian cancer; MPV, mean platelet volume; PFP, platelet-free plasma.

## 2 The coding platelet transcriptome

Platelets are anucleate and possess no available genomic DNA for the transcription of new RNA molecules but contain mitochondrial DNA with the capacity for RNA transcription activity (39). Therefore, most platelet RNAs are either inherited from the transcription of nuclear DNA in the megakaryocyte or acquired by platelets while in circulation (48). Platelets have functional spliceosomes; therefore, they can splice pre-mRNAs into mature mRNA (66). For example, Lindemann et al. (67) reported that interleukin-1β (IL-1β) pre-mRNA was spliced into intronically translatable mRNA in platelets, indicating a broad post-transcriptional regulatory mechanism for platelet mRNA expression. mRNA is the most studied type of RNA in platelets. With the development of high-throughput characterization methods, about one-third of all human genes (~5,000–9,000 genes) transcripts have been identified within platelets (68, 69). Gene ontology (GO) analysis revealed that detectable mRNAs in platelets were enriched in degranulation, coagulation, cytoskeletal dynamics, receptor binding, secretion, etc., which are biological processes closely related to well-known phenotypic activities (70, 71).

Previous studies have illuminated the diagnostic value of platelet mRNA signatures. Nilsson et al. (32) demonstrated that tumor-derived mRNAs were transferred (mutant EGFRvIII) from tumor cells to circulating platelets *in vitro* and *in vivo*. Platelets isolated from glioma and prostate cancer patients contained cancer-related RNA biomarkers EGFRvIII and PCA3, respectively, paving the way for the new potential for cancer diagnostics. Xing et al. (50) described that ITGA2B levels in TEPs were significantly higher in non-small cell lung cancer (NSCLC) patients than in controls, which could be a promising marker to improve the identification of stage I NSCLC patients and distinguish the benign and malignant pulmonary nodules. Interestingly, TIMP1 mRNA was increased in colorectal cancer (CRC) platelets, could be transferred into CRC cells by platelets, and could promote tumor growth *in vivo* and *in vitro* (52). TEP TPM3 mRNA was significantly increased in breast cancer patients, with its transfer into cancer cells mediated by platelet-derived particles to promote cancer cell migration (53). Our lab had identified a higher platelet mRNA expression of apoptotic chromatin coagulation inducing factor 1 (ACIN1) in lung cancer patients than in healthy controls (54), along with a three-platelet mRNA set—MAX, MTURN, and HLA-B—which was significantly upregulated in lung cancer patients processing a dramatically high diagnostic efficiency in female patients; the area under the curve (AUC) was 0.825 (51).

High-throughput RNA sequencing technologies have been employed in platelet RNA profile characterization. For example, the diagnostic potential of TEPs was determined by mRNA sequencing, which could distinguish tumor patients from healthy individuals with 96% accuracy, correctly identified across six different tumor types with 71% accuracy, and also ascertain MET- or HER2-positive and mutant KRAS, EGFR, or PIK3CA tumors (48). Moreover, to select robust biomarker panels for disease classification, the use of “swarm intelligence” was proposed, especially particle swarm optimization (PSO)-enhanced algorithms to analyze differences in RNA splicing isoforms of platelets from patients with NSCLC and healthy volunteers, which could achieve the accurate TEP-based detection of early and advanced NSCLC (37). More recent research has highlighted the potential properties of TEP-derived RNA panels, which correctly detected the presence of cancer in two-thirds of 1,096 blood samples from stage I–IV cancer patients and one-half of 352 stage I–III tumors, with 99% specificity in asymptomatic and 78% specificity in symptomatic controls (72).

## 3 The non-coding platelet transcriptome

Platelets exploit functional spliceosomes, consisting of RNA-binding protein (RBP) and small nuclear RNAs (snRNAs), including U1, U2, U4, U5, and U6. SnRNAs can bind to pre-mRNA to facilitate splicing (43). Interestingly, small nucleolar RNAs (snoRNAs) have also been described as detectable in anucleate platelets (71). SnoRNAs participate in alternative splicing of pre-mRNA in platelets other than regulation of translation in nucleated cells. Some non-coding RNA generations in the platelet also depend on post-transcriptional splicing such as circular RNAs (circRNAs). CircRNAs are generated from mature mRNAs by exonic back-splicing mediated in the spliceosome (73).

Beyond splicing, non-coding RNAs are the second post-transcriptional regulatory mechanism for platelet gene expression, including microRNAs (miRNAs), circRNAs, and long non-coding RNAs (lncRNAs). They can originate from megakaryocytes and also generate in platelets like coding RNA (40). For example, the maturation process of miRNAs in platelets is different from that in nucleated cells. In platelets, miRNA maturation begins with unspliced pre-miRNA, and platelets contain related regulatory proteins Dicer and Argonaute 2 (Ago2), which process pre-miRNA into mature miRNA (74). Non-coding RNAs function in platelets similar to those in nucleated cells; miRNAs destabilize mRNAs and repress translation by harboring 3′-UTR but are sponged by circRNAs. Owing to diverse high-throughput techniques, such as microarrays and RNA-seq, dysregulation of non-coding RNA in TEPs can be easily observed.

### 3.1 MicroRNAs

MiRNAs, a class of small non-coding single-stranded RNAs with approximately 22 nucleotides in length, have highly evolutionarily conserved and tissue-specific expression patterns (75). Decades of research have demonstrated that miRNAs play a crucial role in multiple processes of cancer development. In 2009, Landry et al. (74) confirmed that human platelets contain and release miRNAs, and more than 500 different miRNAs have been identified in human platelets. In addition, human platelet miRNA profiles have extremely high stability (76), which makes platelet miRNA advantageous as diagnostic markers for tumors.

Alteration of platelet miRNA in cancer patients seems to be tumor-specific (77). Wang et al. (55) demonstrated that the expression levels of TEPs miR-34c-3p and miR-18a-5p were significantly higher in patients with nasopharyngeal carcinoma (NPC) compared to healthy subjects. The AUC value of the combined diagnosis of NPC was 0.954. However, this altered expression pattern was not found in plasma miR-34c-3p and miR-18a-5p, suggesting that the aberrances of TEP miR-34c-3p and miR-18a-5p might be the result of the “education” from NPC to platelets. The differential expression of miRNAs in platelets was also observed in a small cohort between pancreatic cancer patients and healthy subjects due to horizontal miRNA transfer between tumors and platelets. Interestingly, this differential miRNA expression was also detected between the blood and pancreatic juice-derived platelets (78). In addition, Diehl et al. (79) reported that miRNAs, including miR-19, miR-21, miR-126, miR-133, miR-146, and miR-223, could be detected in platelet-derived particles, suggesting that platelets could secrete their miRNAs through particles with potential cancer biomarkers. Similarly, the level of miR-223 in platelets of NSCLC patients was higher than in healthy subjects, and platelet-derived particles could effectively deliver miR-223 into human lung cancer cells A549, in which platelet miR-223 targeted EPB41L3 and thus promoted A549 invasion (56).

### 3.2 Circular RNAs

CircRNAs, the class of non-coding RNAs with a structure featuring covalently linked 3′ to 5′ ends, are highly abundant in the human genome (80). Recent studies have shown that circRNAs are differentially expressed in different types of cancer and play a crucial role in several steps of cancer initiation, tumor progression, and drug resistance (81–84). CircRNAs are significantly enriched in platelets 17- to 188-fold relative to nucleated tissues (73, 85), serving as a surrogate marker for mRNA stability in the absence of transcription relative to linear RNAs. Alhasan et al. (73) explained this phenomenon through the degradation/decay of cellular platelet RNA. CircRNAs would be more resistant to degradation by exonucleases. The abundance of circRNAs in platelets relative to megakaryocytes might attribute to circRNA generation in platelets rather than inherit from megakaryocytes (40). Thus, platelet-derived circRNAs may serve as potential novel and promising biomarkers for cancer diagnosis, treatment, and prognosis.

Ambrosi and his colleagues examined the differential circRNA profiles in platelets between NSCLC patients and asymptomatic individuals using high-throughput RNA-seq (49). A total of 4,732 circRNAs were identified, 84 of which were significantly upregulated and 327 were significantly downregulated, suggesting that the platelet circular RNA transcriptome was altered in the presence of cancer. RT-qPCR experiments confirmed that circNRIP1 was downregulated in platelet samples from advanced NSCLC, serving as an indicator of cancer progression. Moreover, a machine learning-based model algorithm was constructed for early-stage lung cancer detection based on combinatorial analysis of blood platelet-derived circRNA and mRNA signature. Combinatorial analysis, including both types of RNAs, resulted in an eight-target signature (six mRNAs and two circRNAs), enhancing the differentiation of lung cancer from controls (AUC of 0.92) (86).

### 3.3 Long non-coding RNA

LncRNA refers to transcripts longer than 200 nucleotides without the protein-coding ability (87). LncRNAs can act as decoys, guides, signals, or scaffolds to combine with DNA, RNA, or proteins to exert various biological functions (40). A large number of studies have shown that abnormal expression of lncRNAs in various types of cancer is associated with cancer recurrence, metastasis, and poor prognosis (88). Sun et al. (89) performed large-scale deep sequencing of human platelets, and a large number of lncRNAs were detected; the lncRNAs in TEPs are rarely reported.

Luo et al. (58) found that the levels of MAGI2-AS3 and ZFAS1 in plasma and platelets of NSCLC patients were significantly downregulated compared to those in healthy controls. Wei et al. (59) found that the TEP lncRNA-ROR of NPC patients was significantly lower than that of healthy subjects, while there was no significant difference in plasma lncRNA-ROR. Ye et al. (60) found that four lncRNA (LNCAROD, SNHG20, LINC00534, and TSPOAP-AS1) were dysregulated in TEPs of CRC patients and could be used as potential diagnostic and discriminative biomarkers for CRC. Our group also identified TEP linc-GTF2H2-1, RP3-466P17.2, and LCC-ST8SIA4-12 as promising biomarkers for NSCLC based on lncRNA microarray and PCR validation (57), suggesting that lncRNAs derived from TEPs can be used in the diagnosis and prediction of cancer progression.

### 3.4 SnRNA and snoRNA

SnRNAs in the spliceosome are not merely the basal factors, ubiquitously expressed in all cells since they are required for post-transcriptional splicing, whereas snRNA levels are extremely variable across a wide range of biological conditions (90). Our lab demonstrated that TEP U1, U2, and U5 were significantly downregulated in lung cancer, which was associated with lung cancer progression, possessing favorable diagnostic efficiencies (61).

The primary function of snoRNAs is not only to guide the epigenetic modification of ribosomal RNAs (rRNAs) (91) but also to mediate pre-mRNA alternative splicing (92). For example, SNORD115 (M/HBII-52) regulated the post-transcriptional processing of serotonin 2C receptor (5-HT2CR) through alternative splicing and control of target mRNA editing (93). The presence of HTR2C pre-mRNA and splicing factors in platelets might indicate that platelet snoRNAs were involved in the mediation of alternative splicing (94). Our group reported that SNORD55 was significantly decreased in TEPs of NSCLC patients, especially of early-stage patients; it exerted a promising diagnostic value for NSCLC with an AUC of 0.803 and also improved the diagnostic accuracy of carcinoembryonic antigen (CEA) for tumor progression (62).

## 4 Platelet proteome

The protein content of platelets can include proteins derived from megakaryocytes, internalized from the extracellular environment, or synthesized within platelets (95). Mature and spliced RNAs can be translated into proteins in the ribosome of platelets.

Tumor cells stimulate platelet activation to release various angiogenic regulatory proteins to promote tumor angiogenesis. Peterson et al. (63) found that vascular endothelial growth factor (VEGF), platelet-derived growth factor (PDGF), and platelet factor 4 (PF4) in platelets of 35 patients with CRC were significantly increased compared to those in 84 healthy controls. Nevertheless, this significant difference was not observed in plasma. Multivariate logistic regression analysis showed that the combined prediction of these three factors for CRC AUC was 0.893. Other studies have found elevated levels of VEGF in platelets in patients with liver cancer (96), lung cancer (97), breast cancer (98), and pancreatic cancer (65).

In recent years, advances in mass spectroscopy-based methods have greatly promoted proteomics research (41). Analysis of platelet protein expression profiles distinguished benign adnexal lesions from International Federation of Gynecology and Obstetrics (FIGO) stage III–IV ovarian cancer, and the multivariate prediction model correctly predicted seven out of eight FIGO stage I–II ovarian cancer cases (64). An analysis of proteomics in patients with early-stage lung cancer (n = 8) and pancreatic cancer (n = 4) found that 85 proteins were significantly altered in platelets in patients with early-stage lung cancer and pancreatic cancer compared to gender- and age-matched controls. After tumor removal, the expression of 81 of the 85 proteins returned to normal levels (99). Multivariate modeling was also performed using six parameters (platelet count, mean platelet volume (MPV), and concentrations of VEGF, PDGF, PF4, CTAPIII, and TSP-1 in platelets and platelet-free plasma (PFP)), and AUC was 0.868 for the diagnosis of lung cancer. The discriminatory ability of the head diagnostic model of pancreatic cancer consisting of three parameters (platelet count, MPV, and VEGF concentration in platelets) to analyze the AUC was 0.827 (65). Taken together, these studies support that platelet-derived proteins can also be used as biomarkers for cancer.

## 5 Conclusion

Early detection of cancer can greatly reduce the probability of distant metastasis, contributing to better treatment outcomes and the quality of life for cancer patients. In recent studies, TEPs appear to be promising candidates as biomarkers for cancer based on liquid biopsies due to the alteration of their transcripts and proteins in response to external signals (100). Platelets are the second most abundant cell in circulation after red blood cells (RBCs) and are easily isolated and counted in blood tests, making them more attractive for clinical applications (8). In recent years, more sensitive new technologies have been developed, such as high-throughput sequencing and mass spectrometry, improving the accuracy and sensitivity of TEP-based liquid biopsies (50).

The unique advantages of platelet RNA and protein in early tumor detection are exciting; however, several challenges still remain to be addressed before they can be applied in clinical trials and practice. All of the studies had small sample sizes that needed to be expanded in further studies. Platelets are easily activated during sample preparation, and the establishment of standardized procedures for TEP research, including pre-analysis processing and specific analysis steps, is far from being implemented so far but is essential and imperative. Moreover, although TEPs are widely recognized as a novel biosource for cancer diagnostics, the mechanisms that tumor educates platelets still remain unclear. Such potential confounding factors should be further addressed in a prospective clinical trial and should be standardized during the blood collection process. Taken together, further characterization of standardized procedures and mechanisms will provide new insights into the diagnostic potential of TEPs and even pave the way for personalized medicine in the future.

## Author contributions

XGS designed and revised the manuscript. QZ wrote the first draft. XGS and XRS reviewed and revised the manuscript. All authors contributed to the article and approved the submitted version.

## References

[1] Fernandez-Lazaro D Garcia Hernandez JL Garcia AC Cordova Martinez A Mielgo-Ayuso J Cruz-Hernandez JJ. Liquid biopsy as novel tool in precision medicine: Origins, properties, identification and clinical perspective of cancer's biomarkers. Diagnostics (Basel). (2020) 10(4):215. doi: 10.3390/diagnostics10040215 32294884 PMC7235853

[2] Gorgannezhad L Umer M Islam MN Nguyen NT Shiddiky MJA. Circulating tumor DNA and liquid biopsy: opportunities, challenges, and recent advances in detection technologies. Lab Chip. (2018) 18(8):1174–96. doi: 10.1039/C8LC00100F 29569666

[3] Economopoulou P Kotsantis I Kyrodimos E Lianidou ES Psyrri A. Liquid biopsy: An emerging prognostic and predictive tool in head and neck squamous cell carcinoma (HNSCC). Focus Circulating Tumor Cells (CTCs). Oral Oncol (2017) 74:83–9. doi: 10.1016/j.oraloncology.2017.09.012 29103757

[4] Di Meo A Bartlett J Cheng Y Pasic MD Yousef GM. Liquid biopsy: a step forward towards precision medicine in urologic malignancies. Mol Cancer. (2017) 16(1):80. doi: 10.1186/s12943-017-0644-5 28410618 PMC5391592

[5] Hassan S Blick T Williams ED Thompson EW. Applications of RNA from circulating tumor cells. Front Biosci (Landmark Ed). (2020) 25(5):874–92. doi: 10.2741/4838 31585921

[6] In 't Veld S Wurdinger T. Tumor-educated platelets. Blood (2019) 133(22):2359–64. doi: 10.1182/blood-2018-12-852830 30833413

[7] Best MG Wesseling P Wurdinger T. Tumor-educated platelets as a noninvasive biomarker source for cancer detection and progression monitoring. Cancer Res (2018) 78(13):3407–12. doi: 10.1158/0008-5472.CAN-18-0887 29921699

[8] Haemmerle M Stone RL Menter DG Afshar-Kharghan V Sood AK. The platelet lifeline to cancer: Challenges and opportunities. Cancer Cell (2018) 33(6):965–83. doi: 10.1016/j.ccell.2018.03.002 PMC5997503 29657130

[9] Koupenova M Clancy L Corkrey HA Freedman JE. Circulating platelets as mediators of immunity, inflammation, and thrombosis. Circ Res (2018) 122(2):337–51. doi: 10.1161/CIRCRESAHA.117.310795 PMC5777300 29348254

[10] Schlesinger M. Role of platelets and platelet receptors in cancer metastasis. J Hematol Oncol (2018) 11(1):125. doi: 10.1186/s13045-018-0669-2 30305116 PMC6180572

[11] Morris K Schnoor B Papa AL. Platelet cancer cell interplay as a new therapeutic target. Biochim Biophys Acta Rev Cancer. (2022) 1877(5):188770. doi: 10.1016/j.bbcan.2022.188770 35926688

[12] Repsold L Pool R Karodia M Tintinger G Joubert AM. An overview of the role of platelets in angiogenesis, apoptosis and autophagy in chronic myeloid leukaemia. Cancer Cell Int (2017) 17:89. doi: 10.1186/s12935-017-0460-4 29118670 PMC5664592

[13] Liu Y Zhang Y Ding Y Zhuang R. Platelet-mediated tumor metastasis mechanism and the role of cell adhesion molecules. Crit Rev Oncol Hematol (2021) 167:103502. doi: 10.1016/j.critrevonc.2021.103502 34662726

[14] Lowe KL Navarro-Nunez L Watson SP. Platelet CLEC-2 and podoplanin in cancer metastasis. Thromb Res (2012) 129 Suppl 1:S30–7. doi: 10.1016/S0049-3848(12)70013-0 22682130

[15] Liu X Song J Zhang H Liu X Zuo F Zhao Y. Immune checkpoint HLA-E:CD94-NKG2A mediates evasion of circulating tumor cells from NK cell surveillance. Cancer Cell (2023) 41(2):272–87e9. doi: 10.1016/j.ccell.2023.01.001 36706761

[16] Maurer S Ferrari de Andrade L. NK cell interaction with platelets and myeloid cells in the tumor milieu. Front Immunol (2020) 11:608849. doi: 10.3389/fimmu.2020.608849 33424862 PMC7785787

[17] Amo L Tamayo-Orbegozo E Maruri N Eguizabal C Zenarruzabeitia O Rinon M. Involvement of platelet-tumor cell interaction in immune evasion. potential role of podocalyxin-like protein 1. Front Oncol (2014) 4:245. doi: 10.3389/fonc.2014.00245 25309871 PMC4160963

[18] Coupland LA Hindmarsh EJ Gardiner EE Parish CR. The influence of platelet membranes on tumour cell behaviour. Cancer Metastasis Rev (2017) 36(2):215–24. doi: 10.1007/s10555-017-9671-3 28707200

[19] Xiong G Chen J Zhang G Wang S Kawasaki K Zhu J. Hsp47 promotes cancer metastasis by enhancing collagen-dependent cancer cell-platelet interaction. Proc Natl Acad Sci U S A. (2020) 117(7):3748–58. doi: 10.1073/pnas.1911951117 PMC7035603 32015106

[20] Wang X Zhao S Wang Z Gao T. Platelets involved tumor cell EMT during circulation: communications and interventions. Cell Commun Signal (2022) 20(1):82. doi: 10.1186/s12964-022-00887-3 35659308 PMC9166407

[21] Lazar S Goldfinger LE. Platelets and extracellular vesicles and their cross talk with cancer. Blood (2021) 137(23):3192–200. doi: 10.1182/blood.2019004119 PMC8351904 33940593

[22] Labelle M Begum S Hynes RO. Direct signaling between platelets and cancer cells induces an epithelial-mesenchymal-like transition and promotes metastasis. Cancer Cell (2011) 20(5):576–90. doi: 10.1016/j.ccr.2011.09.009 PMC3487108 22094253

[23] Razavi AS Mohtashami M Razi S Rezaei N. TGF-beta signaling and the interaction between platelets and T-cells in tumor microenvironment: Foes or friends? Cytokine (2022) 150:155772. doi: 10.1016/j.cyto.2021.155772 34814016

[24] Haschemi R Gockel LM Bendas G Schlesinger M. A combined activity of thrombin and p-selectin is essential for platelet activation by pancreatic cancer cells. Int J Mol Sci (2021) 22(7):3323. doi: 10.3390/ijms22073323 33805059 PMC8037188

[25] Mezouar S Darbousset R Dignat-George F Panicot-Dubois L Dubois C. Inhibition of platelet activation prevents the p-selectin and integrin-dependent accumulation of cancer cell microparticles and reduces tumor growth and metastasis in vivo. Int J Cancer (2015) 136(2):462–75. doi: 10.1002/ijc.28997 24889539

[26] Fabricius HA Starzonek S Lange T. The role of platelet cell surface p-selectin for the direct platelet-tumor cell contact during metastasis formation in human tumors. Front Oncol (2021) 11:642761. doi: 10.3389/fonc.2021.642761 33791226 PMC8006306

[27] Qian W Tao L Wang Y Zhang F Li M Huang S. Downregulation of integrins in cancer cells and anti-platelet properties are involved in holothurian glycosaminoglycan-mediated disruption of the interaction of cancer cells and platelets in hematogenous metastasis. J Vasc Res (2015) 52(3):197–209. doi: 10.1159/000439220 26488158

[28] Zara M Canobbio I Visconte C Canino J Torti M Guidetti GF. Molecular mechanisms of platelet activation and aggregation induced by breast cancer cells. Cell Signal (2018) 48:45–53. doi: 10.1016/j.cellsig.2018.04.008 29705335

[29] Grossi IM Fitzgerald LA Kendall A Taylor JD Sloane BF Honn KV. Inhibition of human tumor cell induced platelet aggregation by antibodies to platelet glycoproteins ib and IIb/IIIa. Proc Soc Exp Biol Med (1987) 186(3):378–83. doi: 10.3181/00379727-186-3-RC1 3423021

[30] Clezardin P Drouin J Morel-Kopp MC Hanss M Kehrel B Serre CM. Role of platelet membrane glycoproteins Ib/IX and IIb/IIIa, and of platelet alpha-granule proteins in platelet aggregation induced by human osteosarcoma cells. Cancer Res (1993) 53(19):4695–700. 7691402

[31] Sabrkhany S Kuijpers MJE Griffioen AW Oude Egbrink MGA. Platelets: the holy grail in cancer blood biomarker research? Angiogenesis (2019) 22(1):1–2. doi: 10.1007/s10456-018-9651-4 30341541

[32] Nilsson RJ Balaj L Hulleman E van Rijn S Pegtel DM Walraven M. Blood platelets contain tumor-derived RNA biomarkers. Blood (2011) 118(13):3680–3. doi: 10.1182/blood-2011-03-344408 PMC7224637 21832279

[33] Lemancewicz D Bolkun L Mantur M Semeniuk J Kloczko J Dzieciol J. Bone marrow megakaryocytes, soluble p-selectin and thrombopoietic cytokines in multiple myeloma patients. Platelets (2014) 25(3):181–7. doi: 10.3109/09537104.2013.805405 23855381

[34] Leblanc R Peyruchaud O. The role of platelets and megakaryocytes in bone metastasis. J Bone Oncol (2016) 5(3):109–11. doi: 10.1016/j.jbo.2016.02.007 PMC5063221 27761368

[35] Downes K Megy K Duarte D Vries M Gebhart J Hofer S. Diagnostic high-throughput sequencing of 2396 patients with bleeding, thrombotic, and platelet disorders. Blood (2019) 134(23):2082–91. doi: 10.1182/blood.2018891192 PMC6993014 31064749

[36] Freson K Turro E. High-throughput sequencing approaches for diagnosing hereditary bleeding and platelet disorders. J Thromb Haemost. (2017) 15(7):1262–72. doi: 10.1111/jth.13681 28671349

[37] Best MG Sol N In 't Veld S Vancura A Muller M Niemeijer AN. Swarm intelligence-enhanced detection of non-Small-Cell lung cancer using tumor-educated platelets. Cancer Cell (2017) 32(2):238–52 e9. doi: 10.1016/j.ccell.2017.07.004 28810146 PMC6381325

[38] Sol N In 't Veld S Vancura A Tjerkstra M Leurs C Rustenburg F. Tumor-educated platelet RNA for the detection and (Pseudo)progression monitoring of glioblastoma. Cell Rep Med (2020) 1(7):100101. doi: 10.1016/j.xcrm.2020.100101 33103128 PMC7576690

[39] Melchinger H Jain K Tyagi T Hwa J. Role of platelet mitochondria: Life in a nucleus-free zone. Front Cardiovasc Med (2019) 6:153. doi: 10.3389/fcvm.2019.00153 31737646 PMC6828734

[40] Gutmann C Joshi A Zampetaki A Mayr M. The landscape of coding and noncoding RNAs in platelets. Antioxid Redox Signal (2021) 34(15):1200–16. doi: 10.1089/ars.2020.8139 32460515

[41] Loosse C Swieringa F Heemskerk JWM Sickmann A Lorenz C. Platelet proteomics: from discovery to diagnosis. Expert Rev Proteomics. (2018) 15(6):467–76. doi: 10.1080/14789450.2018.1480111 29787335

[42] Weyrich AS Lindemann S Tolley ND Kraiss LW Dixon DA Mahoney TM. Change in protein phenotype without a nucleus: translational control in platelets. Semin Thromb Hemost. (2004) 30(4):491–8. doi: 10.1055/s-2004-833484 15354270

[43] Denis MM Tolley ND Bunting M Schwertz H Jiang H Lindemann S. Escaping the nuclear confines: signal-dependent pre-mRNA splicing in anucleate platelets. Cell (2005) 122(3):379–91. doi: 10.1016/j.cell.2005.06.015 PMC4401993 16096058

[44] Neu CT Gutschner T Haemmerle M. Post-transcriptional expression control in platelet biogenesis and function. Int J Mol Sci (2020) 21(20):7614. doi: 10.3390/ijms21207614 33076269 PMC7589263

[45] Sabrkhany S Kuijpers MJE Oude Egbrink MGA Griffioen AW. Platelets as messengers of early-stage cancer. Cancer Metastasis Rev (2021) 40(2):563–73. doi: 10.1007/s10555-021-09956-4 PMC8213673 33634328

[46] Hinterleitner C Strahle J Malenke E Hinterleitner M Henning M Seehawer M. Platelet PD-L1 reflects collective intratumoral PD-L1 expression and predicts immunotherapy response in non-small cell lung cancer. Nat Commun (2021) 12(1):7005. doi: 10.1038/s41467-021-27303-7 34853305 PMC8636618

[47] Wang Z Fang M Li J Yang R Du J Luo Y. High platelet levels attenuate the efficacy of platinum-based treatment in non-small cell lung cancer. Cell Physiol Biochem (2018) 48(6):2456–69. doi: 10.1159/000492683 30121639

[48] Best MG Sol N Kooi I Tannous J Westerman BA Rustenburg F. RNA-Seq of tumor-educated platelets enables blood-based pan-cancer, multiclass, and molecular pathway cancer diagnostics. Cancer Cell (2015) 28(5):666–76. doi: 10.1016/j.ccell.2015.09.018 PMC4644263 26525104

[49] D’Ambrosi S Visser A Antunes-Ferreira M Poutsma A Giannoukakos S Sol N. The analysis of platelet-derived circRNA repertoire as potential diagnostic biomarker for non-small cell lung cancer. Cancers (2021) 13(18):4644. doi: 10.3390/cancers13184644 34572871 PMC8468408

[50] Xing S Zeng T Xue N He Y Lai YZ Li HL. Development and validation of tumor-educated blood platelets integrin alpha 2b (ITGA2B) RNA for diagnosis and prognosis of non-small-cell lung cancer through RNA-seq. Int J Biol Sci (2019) 15(9):1977–92. doi: 10.7150/ijbs.36284 PMC6743295 31523198

[51] Liu L Song X Li X Xue L Ding S Niu L. A three-platelet mRNA set: MAX, MTURN and HLA-b as biomarker for lung cancer. J Cancer Res Clin Oncol (2019) 145(11):2713–23. doi: 10.1007/s00432-019-03032-9 PMC11810211 31552488

[52] Yang L Jiang Q Li DZ Zhou X Yu DS Zhong J. TIMP1 mRNA in tumor-educated platelets is diagnostic biomarker for colorectal cancer. Aging (Albany NY). (2019) 11(20):8998–9012. doi: 10.18632/aging.102366 31639773 PMC6834400

[53] Yao B Qu S Hu R Gao W Jin S Ju J. Delivery of platelet TPM3 mRNA into breast cancer cells *via* microvesicles enhances metastasis. FEBS Open Bio. (2019) 9(12):2159–69. doi: 10.1002/2211-5463.12759 PMC6886296 31705785

[54] Xue L Xie L Song X Song X. [Expression and significance of ACIN1 mRNA in platelets of lung cancer]. Zhongguo Fei Ai Za Zhi. (2018) 21(9):677–81. doi: 10.3779/j.issn.1009-3419.2018.09.05 PMC6137000 30201066

[55] Wang H Wei X Wu B Su J Tan W Yang K. Tumor-educated platelet miR-34c-3p and miR-18a-5p as potential liquid biopsy biomarkers for nasopharyngeal carcinoma diagnosis. Cancer Manag Res (2019) 11:3351–60. doi: 10.2147/CMAR.S195654 PMC6489554 31114371

[56] Liang H Yan X Pan Y Wang Y Wang N Li L. MicroRNA-223 delivered by platelet-derived microvesicles promotes lung cancer cell invasion *via* targeting tumor suppressor EPB41L3. Mol Cancer. (2015) 14:58. doi: 10.1186/s12943-015-0327-z 25881295 PMC4360939

[57] Li X Liu L Song X Wang K Niu L Xie L. TEP linc-GTF2H2-1, RP3-466P17.2, and lnc-ST8SIA4-12 as novel biomarkers for lung cancer diagnosis and progression prediction. J Cancer Res Clin Oncol (2021) 147(6):1609–22. doi: 10.1007/s00432-020-03502-5 PMC11801899 33792796

[58] Luo CL Xu ZG Chen H Ji J Wang YH Hu W. LncRNAs and EGFRvIII sequestered in TEPs enable blood-based NSCLC diagnosis. Cancer Manag Res (2018) 10:1449–59. doi: 10.2147/CMAR.S164227 PMC5997181 29922089

[59] Wei J Meng X Wei X Zhu K Du L Wang H. Down-regulated lncRNA ROR in tumor-educated platelets as a liquid-biopsy biomarker for nasopharyngeal carcinoma. J Cancer Res Clin Oncol (2022). doi: 10.1007/s00432-022-04350-1 PMC10349751 36107245

[60] Ye B Li F Chen M Weng Y Qi C Xie Y. A panel of platelet-associated circulating long non-coding RNAs as potential biomarkers for colorectal cancer. Genomics (2022) 114(1):31–7. doi: 10.1016/j.ygeno.2021.11.026 34843904

[61] Dong X Ding S Yu M Niu L Xue L Zhao Y. Small nuclear RNAs (U1, U2, U5) in tumor-educated platelets are downregulated and act as promising biomarkers in lung cancer. Front Oncol (2020) 10:1627. doi: 10.3389/fonc.2020.01627 32903345 PMC7434840

[62] Dong X Song X Ding S Yu M Shang X Wang K. Tumor-educated platelet SNORD55 as a potential biomarker for the early diagnosis of non-small cell lung cancer. Thorac Cancer. (2021) 12(5):659–66. doi: 10.1111/1759-7714.13823 PMC7919130 33474827

[63] Peterson JE Zurakowski D Italiano JE Jr. Michel LV Connors S Oenick M. VEGF, PF4 and PDGF are elevated in platelets of colorectal cancer patients. Angiogenesis (2012) 15(2):265–73. doi: 10.1007/s10456-012-9259-z 22402885

[64] Lomnytska M Pinto R Becker S Engstrom U Gustafsson S Bjorklund C. Platelet protein biomarker panel for ovarian cancer diagnosis. biomark Res (2018) 6:2. doi: 10.1186/s40364-018-0118-y 29344361 PMC5767003

[65] Sabrkhany S Kuijpers MJE van Kuijk SMJ Sanders L Pineda S Olde Damink SWM. A combination of platelet features allows detection of early-stage cancer. Eur J Cancer. (2017) 80:5–13. doi: 10.1016/j.ejca.2017.04.010 28527393

[66] Papasaikas P Valcarcel J. The spliceosome: The ultimate RNA chaperone and sculptor. Trends Biochem Sci (2016) 41(1):33–45. doi: 10.1016/j.tibs.2015.11.003 26682498

[67] Lindemann S Tolley ND Dixon DA McIntyre TM Prescott SM Zimmerman GA. Activated platelets mediate inflammatory signaling by regulated interleukin 1beta synthesis. J Cell Biol (2001) 154(3):485–90. doi: 10.1083/jcb.200105058 PMC2196422 11489912

[68] Gnatenko DV Dunn JJ Schwedes J Bahou WF. Transcript profiling of human platelets using microarray and serial analysis of gene expression (SAGE). Methods Mol Biol (2009) 496:245–72. doi: 10.1007/978-1-59745-553-4_16 18839115

[69] Bugert P Dugrillon A Gunaydin A Eichler H Kluter H. Messenger RNA profiling of human platelets by microarray hybridization. Thromb Haemost. (2003) 90(4):738–48. doi: 10.1055/s-0037-1613622 14515197

[70] Mills EW Green R Ingolia NT. Slowed decay of mRNAs enhances platelet specific translation. Blood (2017) 129(17):e38–48. doi: 10.1182/blood-2016-08-736108 PMC5409447 28213379

[71] Bray PF McKenzie SE Edelstein LC Nagalla S Delgrosso K Ertel A. The complex transcriptional landscape of the anucleate human platelet. BMC Genomics (2013) 14:1. doi: 10.1186/1471-2164-14-1 23323973 PMC3722126

[72] In 't Veld S Arkani M Post E Antunes-Ferreira M D'Ambrosi S Vessies DCL. Detection and localization of early- and late-stage cancers using platelet RNA. Cancer Cell (2022) 40(9):999–1009 e6. doi: 10.1016/j.ccell.2022.08.006 36055228

[73] Alhasan AA Izuogu OG Al-Balool HH Steyn JS Evans A Colzani M. Circular RNA enrichment in platelets is a signature of transcriptome degradation. Blood (2016) 127(9):e1–e11. doi: 10.1182/blood-2015-06-649434 26660425 PMC4797142

[74] Landry P Plante I Ouellet DL Perron MP Rousseau G Provost P. Existence of a microRNA pathway in anucleate platelets. Nat Struct Mol Biol (2009) 16(9):961–6. doi: 10.1038/nsmb.1651 PMC2911476 19668211

[75] Saliminejad K Khorram Khorshid HR Soleymani Fard S Ghaffari SH. An overview of microRNAs: Biology, functions, therapeutics, and analysis methods. J Cell Physiol (2019) 234(5):5451–65. doi: 10.1002/jcp.27486 30471116

[76] Stratz C Nuhrenberg TG Binder H Valina CM Trenk D Hochholzer W. Micro-array profiling exhibits remarkable intra-individual stability of human platelet micro-RNA. Thromb Haemost. (2012) 107(4):634–41. doi: 10.1160/TH11-10-0742 22371016

[77] Sol N Wurdinger T. Platelet RNA signatures for the detection of cancer. Cancer Metastasis Rev (2017) 36(2):263–72. doi: 10.1007/s10555-017-9674-0 PMC5557864 28681241

[78] Diaz-Blancas JY Dominguez-Rosado I Chan-Nunez C Melendez-Zajgla J Maldonado V. Pancreatic cancer cells induce MicroRNA deregulation in platelets. Int J Mol Sci (2022) 23(19):11438. doi: 10.3390/ijms231911438 36232741 PMC9569638

[79] Diehl P Fricke A Sander L Stamm J Bassler N Htun N. Microparticles: major transport vehicles for distinct microRNAs in circulation. Cardiovasc Res (2012) 93(4):633–44. doi: 10.1093/cvr/cvs007 PMC3291092 22258631

[80] Patop IL Wust S Kadener S. Past, present, and future of circRNAs. EMBO J (2019) 38(16):e100836. doi: 10.15252/embj.2018100836 31343080 PMC6694216

[81] Yin Y Long J He Q Li Y Liao Y He P. Emerging roles of circRNA in formation and progression of cancer. J Cancer. (2019) 10(21):5015–21. doi: 10.7150/jca.30828 PMC6775606 31602252

[82] Sarkar D Diermeier SD. Circular RNAs: Potential applications as therapeutic targets and biomarkers in breast cancer. Noncoding RNA. (2021) 7(1):2. doi: 10.3390/ncrna7010002 33466455 PMC7838774

[83] Micallef I Baron B. The mechanistic roles of ncRNAs in promoting and supporting chemoresistance of colorectal cancer. Noncoding RNA. (2021) 7(2):24. doi: 10.3390/ncrna7020024 33807355 PMC8103280

[84] Bach DH Lee SK Sood AK. Circular RNAs in cancer. Mol Ther Nucleic Acids (2019) 16:118–29. doi: 10.1016/j.omtn.2019.02.005 PMC6411617 30861414

[85] Preusser C Hung LH Schneider T Schreiner S Hardt M Moebus A. Selective release of circRNAs in platelet-derived extracellular vesicles. J Extracell Vesicles. (2018) 7(1):1424473. doi: 10.1080/20013078.2018.1424473 29359036 PMC5769804

[86] D'Ambrosi S Giannoukakos S Antunes-Ferreira M Pedraz-Valdunciel C Bracht JWP Potie N. Combinatorial blood platelets-derived circRNA and mRNA signature for early-stage lung cancer detection. Int J Mol Sci (2023) 24(5):4881. doi: 10.3390/ijms24054881 36902312 PMC10003255

[87] Spizzo R Almeida MI Colombatti A Calin GA. Long non-coding RNAs and cancer: a new frontier of translational research? Oncogene (2012) 31(43):4577–87. doi: 10.1038/onc.2011.621 PMC3433647 22266873

[88] Chi Y Wang D Wang J Yu W Yang J. Long non-coding RNA in the pathogenesis of cancers. Cells (2019) 8(9):1015. doi: 10.3390/cells8091015 31480503 PMC6770362

[89] Sun Y Liu R Xia X Xing L Jiang J Bian W. Large-Scale profiling on lncRNAs in human platelets: Correlation with platelet reactivity. Cells (2022) 11(14):2256. doi: 10.3390/cells11142256 35883699 PMC9319970

[90] Dvinge H Guenthoer J Porter PL Bradley RK. RNA Components of the spliceosome regulate tissue- and cancer-specific alternative splicing. Genome Res (2019) 29(10):1591–604. doi: 10.1101/gr.246678.118 PMC6771400 31434678

[91] Jorjani H Kehr S Jedlinski DJ Gumienny R Hertel J Stadler PF. An updated human snoRNAome. Nucleic Acids Res (2016) 44(11):5068–82. doi: 10.1093/nar/gkw386 PMC4914119 27174936

[92] Falaleeva M Pages A Matuszek Z Hidmi S Agranat-Tamir L Korotkov K. Dual function of C/D box small nucleolar RNAs in rRNA modification and alternative pre-mRNA splicing. Proc Natl Acad Sci U S A. (2016) 113(12):E1625–34. doi: 10.1073/pnas.1519292113 PMC4812717 26957605

[93] Kishore S Khanna A Zhang Z Hui J Balwierz PJ Stefan M. The snoRNA MBII-52 (SNORD 115) is processed into smaller RNAs and regulates alternative splicing. Hum Mol Genet (2010) 19(7):1153–64. doi: 10.1093/hmg/ddp585 PMC2838533 20053671

[94] Best MG Vancura A Wurdinger T. Platelet RNA as a circulating biomarker trove for cancer diagnostics. J Thromb Haemost. (2017) 15(7):1295–306. doi: 10.1111/jth.13720 28671345

[95] Zufferey A Schvartz D Nolli S Reny JL Sanchez JC Fontana P. Characterization of the platelet granule proteome: evidence of the presence of MHC1 in alpha-granules. J Proteomics. (2014) 101:130–40. doi: 10.1016/j.jprot.2014.02.008 24549006

[96] Kim SJ Choi IK Park KH Yoon SY Oh SC Seo JH. Serum vascular endothelial growth factor per platelet count in hepatocellular carcinoma: correlations with clinical parameters and survival. Jpn J Clin Oncol (2004) 34(4):184–90. doi: 10.1093/jjco/hyh039 15121753

[97] Wiesner T Bugl S Mayer F Hartmann JT Kopp HG. Differential changes in platelet VEGF, tsp, CXCL12, and CXCL4 in patients with metastatic cancer. Clin Exp Metastasis. (2010) 27(3):141–9. doi: 10.1007/s10585-010-9311-6 20182908

[98] Holmes CE Levis JE Schneider DJ Bambace NM Sharma D Lal I. Platelet phenotype changes associated with breast cancer and its treatment. Platelets (2016) 27(7):703–11. doi: 10.3109/09537104.2016.1171302 27135253

[99] Sabrkhany S Kuijpers MJE Knol JC Olde Damink SWM Dingemans AC Verheul HM. Exploration of the platelet proteome in patients with early-stage cancer. J Proteomics. (2018) 177:65–74. doi: 10.1016/j.jprot.2018.02.011 29432918

[100] Antunes-Ferreira M Koppers-Lalic D Wurdinger T. Circulating platelets as liquid biopsy sources for cancer detection. Mol Oncol (2021) 15(6):1727–43. doi: 10.1002/1878-0261.12859 PMC8169446 33219615

